# Acute interstitial nephritis with podocyte foot-process effacement complicating *Plasmodium falciparum* infection

**DOI:** 10.1186/s12936-019-2674-5

**Published:** 2019-03-01

**Authors:** Patrick J. Gleeson, John A. O’Regan, Teresa McHale, Helen Tuite, Louise Giblin, Donal Reddan

**Affiliations:** 1 Department of Nephrology, University College Hospital, Galway, Republic of Ireland; 2 Department of Pathology, University College Hospital, Galway, Republic of Ireland; 3 Department of Infectious Disease, University College Hospital, Galway, Republic of Ireland; 4 Immune Receptors and Renal Immunopathology, INSERM Unit 1149, Centre de Recherche sur l’Inflammation, Université Sorbonne Paris Cité, Paris, France

**Keywords:** Malarial acute renal failure (MARF), Acute interstitial nephritis, Minimal change disease (MCD), Podocyte, Severe malaria

## Abstract

**Background:**

Malarial acute renal failure (MARF) is a component of the severe malaria syndrome, and complicates 1–5% of malaria infections. This form of renal failure has not been well characterized by histopathology.

**Case presentation:**

A 44 year-old male presented to the emergency department with a 5-day history of fever and malaise after returning from Nigeria. A blood film was positive for *Plasmodium falciparum*. His creatinine was 616 µmol/L coming from a normal baseline of 89 µmol/L. He had a urine protein:creatinine ratio of 346 mg/mmol (4.4 g/L). He required dialysis. A renal biopsy showed acute interstitial nephritis with podocyte foot-process effacement. He was treated with artesunate and his renal function improved. At 1 year follow-up his creatinine had plateaued at 120 µmol/L with persistent low-grade proteinuria.

**Conclusion:**

Acute interstitial nephritis and podocyte foot-process effacement might be under-recognized lesions in MARF. Studying the mechanisms of MARF could give insight into the immunopathology of severe malaria.

## Background

Acute renal failure complicates 1–5% of malaria infections and this syndrome is referred to as malarial acute renal failure (MARF). MARF is a criterion for severe malaria and its associated mortality rate is 15–45% [1, 2]. *Plasmodium falciparum* is nearly always the infecting pathogen in MARF; rarely, *Plasmodium vivax* has been implicated [2, 3]. MARF has not been well characterized by histopathological examination of renal biopsies. Sparse clinical and animal data suggest that the underlying renal pathologies include acute tubular necrosis (ATN), post-infectious glomerulonephritis and mesangio-proliferative glomerulonephritis [2, 4]. Acute interstitial nephritis (AIN) has only been described in animal models of malaria [2, 5]. Recognized pathogenic mechanisms of MARF include sequestration of parasitized erythrocytes in the renal microcirculation resulting in ATN [6], endothelial activation [7] and toxicity from free haemoglobin [8]. Proteinuria is a frequent feature of *P. falciparum* infection [9, 10] however, the lesions responsible for this are not clear, as electron microscopy studies of affected glomeruli have not been reported. Further delineating the mechanisms of MARF in humans could inform treatment strategies and improve our understanding of the inflammatory response to malaria, particularly in severe malaria. Here, a case of MARF with histologically proven eosinophilic AIN and podocyte foot-process effacement is reported.

## Case

A 44 year-old male presented to the emergency department with a 5-day history of fever and malaise. He had recently returned to Ireland (his country of residence for 10 years) from Nigeria (his native country) after visiting friends and relatives, without taking malaria prophylaxis. He had a history of hypertension, for which he took ramipril, amlodipine and bendroflumethiazide throughout the previous year. There was no family history of renal disease. He reported having taken over the counter paracetamol during the 5 days prior to presentation, and a single 400 mg dose of ibuprofen on the day of presentation. Consumption of non-steroidal anti-inflammatory drugs (NSAIDs) beyond the day of presentation was repeatedly denied. He had not taken any other medications commonly associated with AIN such as beta-lactams, fluoroquinolones, sulfonamides or proton pump inhibitors prior to presentation.

On examination, he was euvolaemic, his blood pressure was 169/77 mmHg and he produced 1580 mls of dark urine during the first 24 h. Urinalysis revealed 4+ protein and 3+ blood. He did not have a rash and had no peripheral oedema.

Initial routine blood tests included creatinine 616 µmol/L (baseline 89 µmol/L, 5 months before presentation), haemoglobin 11.2 g/dL, platelet count 70 × 10^9^/L, eosinophil count 0.1 × 10^9^/L, serum albumin 26 g/L, total serum bilirubin 15 μmol/L and lactate dehydrogenase 960 U/L. A blood film was positive for *P. falciparum* with 0.4% parasitaemia. Initial urine protein–creatinine ratio was 346 mg/mmol (absolute proteinuria = 4448 mg/L).

Tests for HIV, HBV, HCV, ANA and ANCA were all negative. C3 was normal and C4 was low (0.09 g/L). His haptoglobin was low (0.24 g/L) and G6PD enzyme activity was normal. A renal ultrasound described diffusely echogenic kidneys with the right kidney measuring 130 mm and the left kidney measuring 143 mm.

**Fig. 1:**
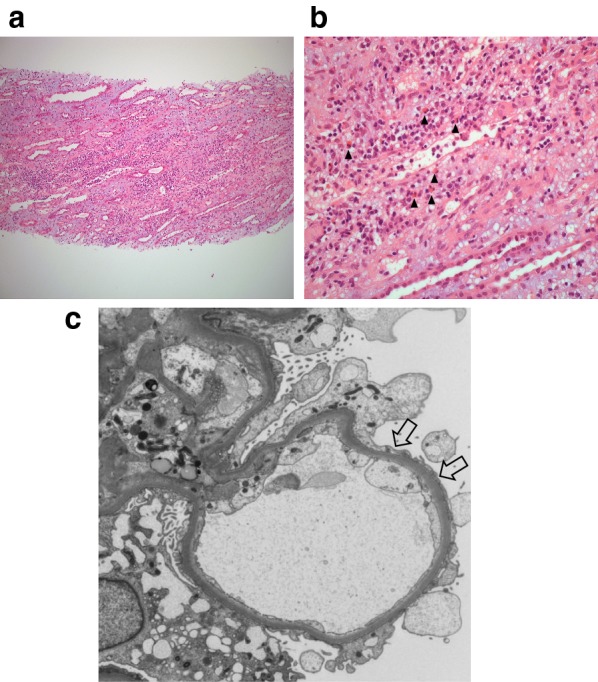
**a** H&E stain of the patient’s renal biopsy demonstrating an interstitial inflammatory infiltrate (**b**) with numerous eosinophils (arrowheads); **c** Electron microscopy image showing an open capillary loop with surrounding podocyte foot-process effacement (arrows) (magnification × 3000)

A renal biopsy performed 10 days after presentation demonstrated acute interstitial nephritis with numerous eosinophils, particularly at the cortico-medullary junction (Fig. 1a). There was an absence of neutrophils or granulomas. Immunofluorescence staining showed no specific pattern of antibody deposition for standard antisera (anti-IgG, anti-IgA, anti-IgM, anti-C3, anti-kappa, anti-lambda, anti-fibrin were all negative). Interstitial fibrosis was minimal. Electron microscopy revealed podocyte foot-process fusion involving the majority of capillaries, and the majority of the surface of affected capillaries, with microvillous transformation of the podocyte cytoplasm (Fig. 1b).

**Fig. 2:**
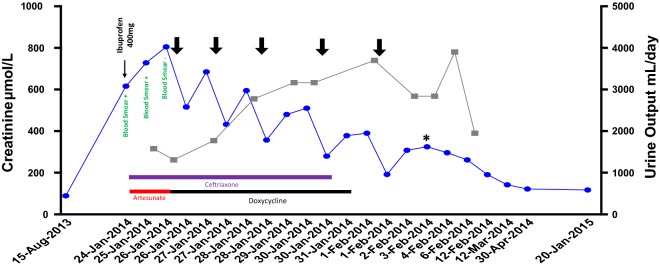
Timeline of patient’s creatinine (blue) from time of normal measurement 5-months prior to admission to 1-year follow up, and urine output (grey) during admission. The timing of significant medications are indicated. Haemodialysis sessions are indicated by bold arrows. The day of renal biopsy is indicated by an asterisk (*)

The patient was treated for severe malaria with intravenous artesunate on day 1, followed by a further 3 days of artesunate and a further 7 days of oral doxycycline. He was also covered empirically with ceftriaxone. Intermittent haemodialysis was started on hospital-day 3, as renal function was not recovering and the patient developed symptoms of uraemia. He received five sessions of intermittent haemodialysis before regaining independent renal function (Fig. 2). At 1 year follow up his creatinine had plateaued around 120 µmol/L, with persistent proteinuria (protein:creatinine ratio 172 mg/mmol) after restarting ramipril, amlodipine and bendroflumethiazide.

## Discussion

Two important renal lesions were identified in this patient with MARF; the presence of eosinophilic AIN explains the acute drop in glomerular filtration rate, and fusion of the podocyte foot-processes (“minimal change disease”, MCD) explains the heavy proteinuria. The relationship between these two lesions is unclear, although they both likely arose through the same inflammatory process. Parasitic infections can have a causative role in both AIN [11] and MCD [12, 13] however, the two typically arise together in drug-induced disease [14, 15].

A critical point in this case is to discern whether the interstitial nephritis was triggered by a drug or by the infecting pathogen. The patient had normal renal function documented while taking his anti-hypertensives, and there was no relapse after re-challenge with these medications. His renal failure was well established at presentation and renal function began to improve during his admission, so the medications he received in hospital cannot be held accountable. The consumption of a non-steroidal anti-inflammatory drug (NSAID) mandates further discussion.

NSAID induced interstitial nephritis occurs 2 weeks to 18 months after initial exposure to the drug [15]. A lymphocytic interstitial infiltrate predominates and there can be associated glomerular changes, such as minimal change or membranous nephropathy. NSAID induced disease is not associated with typical stigmata of an allergic reaction such as fever, rash, eosinophilia or eosinophiluria, and the proteinuria typically remits on withdrawal of the offending drug [15, 16]. The patient was only exposed to an NSAID on the day of presentation, which was too soon to explain his acute kidney injury, the biopsy showed an eosinophilic infiltrate and he has persistent proteinuria, so the renal findings cannot be explained by his drug history, which was corroborated a number of different times by different doctors.

Reports of renal biopsies from patients with MARF are scarce and mostly originate from India [17, 18]. One biopsy series of 20 patients with MARF found either ATN, mesangioproliferative glomerulonephritis or a combination of both [17]. Prakash et al. [18] reported a series of 6 MARF biopsies which revealed 1 necrotising glomerulonephritis while the other five had ATN. Owl monkeys challenged with *P. falciparum* were found to have renal malaria antigen deposition and interstitial nephritis [5].

Many infective organisms have been associated with interstitial nephritis [19], including a case of AIN associated with *Babesia microti*, a protozoan closely related to *Plasmodium*. In this case the inflammatory infiltrate also contained eosinophils and there was focal podocyte foot-process effacement [20], very similar to the present case.

Proteinuria is not uncommon in malaria infection [9, 10]. Ehrich et al. [9] reported proteinuria in 44% of patients with *P. falciparum* infection ranging from 0.15 to 5 g/day; there were no accompanying renal biopsies and, based on urine electrophoresis, they concluded that both glomerular and tubular sources could account for the proteinuria. More recently Ogbadoyi et al. [10] reported from Nigeria that about 70% of malaria patients had proteinuria, typically without an associated rise in creatinine, but again without any renal biopsy to explain the source of proteinuria. The podocyte foot-process fusion seen on electron microscopy explains the severe proteinuria seen in this patient; a similar phenomenon, with or without AIN, could explain the glomerular proteinuria seen in some other patients with *P. falciparum* infection.

MARF is predominantly a disease of partially- or non-immune adults in low-transmission endemic regions [1]—a population found mostly in Asia, which explains the high representation of published case-series from this region. In Africa, home to the greatest burden of *P. falciparum*, malaria is overwhelmingly a disease of children, in whom MARF is unusual [21]. However, given the volume of children infected, malaria is still an important cause of paediatric AKI and paediatric dialysis in sub-Saharan Africa [22], and MARF appears to be on the increase among children [1]. Malaria was the third greatest cause of AKI and dialysis at a paediatric teaching hospital in Nigeria [22]. A limitation of resources in sub-Saharan Africa, precluding detailed histological renal investigation, may have led to under-recognition of AIN and MCD as complications of *P. falciparum* infection. Given its absence in Asian case-series however, AIN is unlikely to be a common cause of MARF.

People living in malaria endemic regions develop an asymptomatic, non-sterile immunity to malaria after repeated exposure to infection, known as “premunition” [23, 24]. It is in the parasite’s interest not to kill its host, and the development of severe malaria might result from a dysregulated immune response to the parasite rather than from parasite virulence [25, 26]. The highest incidence of imported malaria in Europe is amongst immigrants native to malaria-endemic regions that return after visiting friends and relatives (VFR) [27]. This group are less inclined to take malaria prophylaxis as they do not expect to succumb to malaria however, premunition wanes after about 1 year of non-exposure to malaria antigen [24, 28]. This partially-immune VFR group are less likely to develop severe malaria than their European-tourist counterparts [26] but, anecdotally, they tend to develop more severe illness than if they had not left the endemic zone. One possible explanation for this is that they lose immune-tolerance to malaria antigens. Regulatory T cells (Tregs) dampen the immune response to frequently encountered antigens, however memory Tregs have a shorter life-span than memory T helper cells and memory B cells [29]. Experimental evidence of regulation of the anti-malarial immune response by regulatory T cells is accumulating, and these effects may even be organ specific [30]. Interestingly, Treg deficiencies are seen in MCD and immune checkpoint-inhibitor drugs, which counteract immune-tolerance, have been reported to cause MCD [31]. The Th2 immune response and IL-13, which recruit eosinophils to the site of inflammation, have been incriminated in the immunopathology of MCD [13].

The patient had no prior history of renal failure associated with malaria infection, despite coming from an endemic-region. He could have suffered interstitial nephritis with associated podocyte foot-process effacement on this occasion due to a loss of tolerance to malaria, leading to an unbridled cell-mediated inflammatory response to *P. falciparum* antigen deposited in his kidney. *Plasmodium falciparum* antigen deposition in renal tubules has previously been described in a post-mortem study [5]. The local release of cytokines, such as IL-13, could have induced podocyte foot-process effacement or, conversely, an increased leak of antigens through the glomeruli could have triggered a tubulo-interstitial infiltrate.

The low level of C4 in this patient can be attributed to activation of the classical complement pathway by malaria infection [32] and the blood on the urine dipstick is explained by intravascular haemolysis.

Regarding potential treatment options, corticosteroids have been shown to accelerate the rate of recovery from AIN, however they have not been shown to improve long term outcome [33]. Corticosteroids were not given to this patient, as they may be deleterious in acute malaria infection [2]; instead, the underlying cause was targeted with artesunate [34].

## Conclusion

The conclusion that this case of AIN was caused by *P. falciparum* infection is supported firstly by the biological precedent in animal models, secondly, because the natural history of the illness and all clinical features are best explained by the malaria infection and, thirdly, because NSAID induced nephropathy does not fit with the patient’s drug history, histology or outcome. The principles of managing MARF include effective treatment of the infection according to WHO guidelines [35], avoidance of nephrotoxins, optimization of volume status, and initiation of renal replacement therapy when clinically indicated.

Describing the underlying renal pathology found in MARF is important as it can inform treatment strategies and improve our understanding of immunopathology in severe malaria.

## Abbreviations

MARF: malarial acute renal failure
AKI: acute kidney injury
ATN: acute tubular necrosis
AIN: acute interstitial nephritis
HIV: human immunodeficiency virus
HBV: hepatitis B virus
HCV: hepatitis C virus
ANA: anti-nuclear antibody
ANCA: anti-neutrophil cytoplasmic antibody
C3: complement component 3
C4: complement component 4
IgG: immunoglobulin G
IgA: immunoglobulin A
IgM: immunoglobulin M
MCD: minimal change disease
NSAID: non-steroidal anti-inflammatory drug
H&E: haematoxylin and eosin
VFR: visiting friends and relative
